# Short-Term Amygdala Low-Frequency Stimulation Does not Influence Hippocampal Interneuron Changes Observed in the Pilocarpine Model of Epilepsy

**DOI:** 10.3390/cells10030520

**Published:** 2021-03-01

**Authors:** István Mihály, Tímea Molnár, Ádám-József Berki, Réka-Barbara Bod, Károly Orbán-Kis, Zsolt Gáll, Tibor Szilágyi

**Affiliations:** 1 Department of Physiology, Faculty of Medicine, George Emil Palade University of Medicine, Pharmacy, Science, and Technology of Târgu Mureș, 540142 Târgu Mureș, Romania; molnar.timea.orsolya@gmail.com (T.M.); berki.adam@yahoo.com (Á.-J.B.); bod.reka@yahoo.com (R.-B.B.); karoly.orban-kis@umfst.ro (K.O.-K.); tibor.szilagyi@umfst.ro (T.S.); 2 Department of Pharmacology and Clinical Pharmacy, George Emil Palade University of Medicine, Pharmacy, Science, and Technology of Târgu Mureș, 540142 Târgu Mureș, Romania; zsolt.gall@umfst.ro

**Keywords:** temporal lobe epilepsy, pilocarpine, interneurons, amygdala, deep brain stimulation, hippocampus, parvalbumin, neuropeptide Y, neuronal nitric oxide synthase

## Abstract

Temporal lobe epilepsy (TLE) is characterized by changes in interneuron numbers in the hippocampus. Deep brain stimulation (DBS) is an emerging tool to treat TLE seizures, although its mechanisms are not fully deciphered. We aimed to depict the effect of amygdala DBS on the density of the most common interneuron types in the CA1 hippocampal subfield in the lithium-pilocarpine model of epilepsy. Status epilepticus was induced in male Wistar rats. Eight weeks later, a stimulation electrode was implanted to the left basolateral amygdala of both pilocarpine-treated (Pilo, *n* = 14) and age-matched control rats (*n* = 12). Ten Pilo and 4 control animals received for 10 days 4 daily packages of 50 s 4 Hz regular stimulation trains. At the end of the stimulation period, interneurons were identified by immunolabeling for parvalbumin (PV), neuropeptide Y (NPY), and neuronal nitric oxide synthase (nNOS). Cell density was determined in the CA1 subfield of the hippocampus using confocal microscopy. We found that PV+ cell density was preserved in pilocarpine-treated rats, while the NPY+/nNOS+ cell density decreased significantly. The amygdala DBS did not significantly change the cell density in healthy or in epileptic animals. We conclude that DBS with low frequency applied for 10 days does not influence interneuron cell density changes in the hippocampus of epileptic rats.

## 1. Introduction

Epilepsy is a widespread neurological disease, affecting more than 1% of the world’s population [1], representing an essential socio-economic burden to patients and society [2]. A frequent epilepsy type is temporal lobe epilepsy (TLE), characterized by recurrent, spontaneous seizures originating from temporal lobe structures, especially from the hippocampus [3,4]. It is currently incurable, and around 20 to 40% of patients are resistant to existing antiepileptic drugs [5]. In advanced stages of the disease, marked neuronal cell death and volume shrinkage occur in hippocampal CA1 and CA3 areas called hippocampal sclerosis [6]. A complex morphofunctional reorganization precedes sclerosis: cell death, axonal sprouting, and neurogenesis also occur [7,8,9]. GABAergic interneurons represent a heterogeneous population, with many different subtypes defined by their cell body’s location, protein expression, electrophysiological pattern, subcellular localization of their axonal projection to the postsynaptic cell, etc. [10,11]. Their contribution to normal network activity is crucial by modulating excitability, threshold potential, synchrony, and temporal regulation of pyramidal cell activity [12]. GABAergic cells are vulnerable to epileptic insult, and changes in their numbers and/or function can lead to an imbalance between inhibition and excitation in hippocampal circuitry and promote epileptogenesis [13,14,15,16,17]. The CA1 area of the hippocampus is one of the most studied brain regions. Its intrinsic structure is relatively well-known, and it has at least 23 inhibitory cell types [18,19].

Interneurons expressing the protein parvalbumin (PV) and those co-expressing neuropeptide Y (NPY) and neuronal nitric oxide synthase (nNOS) represent the most numerous groups of interneurons in this area [15,19,20]. Parvalbumin-containing cells are basket, bistratified, oriens-lacunosum molecular (O-LM), and axo-axonic cells [11]. Those expressing both PV and NPY are bistratified cells [21]. Loss of PV+ cells in the epileptic human and rodent hippocampus was already reported [22,23], but sparing of their density was also reported [24].

The cells co-expressing NPY and nNOS are the so-called ivy and neurogliaform cells. They produce nitrogen oxide and NPY, and besides having a role in GABAergic inhibition, they are involved in modulating general excitability and synaptic plasticity, too [25,26,27]. In TLE, the number of nNOS+ cells is decreased in both humans and rats [24,28,29].

For a long time, surgical removal of epileptic tissue was the only option for patients who could not be appropriately treated with antiepileptic drugs. Still, postoperative complications are numerous, and around 40% will not be seizure-free [30,31]. In recent years electrical stimulation of deep brain structures (deep brain stimulation, DBS) with surgically implanted electrodes emerged as an alternative therapeutic tool [32] and effectively reduced seizure numbers in a substantial proportion of TLE patients [33,34,35]. DBS also showed anticonvulsant action in status epilepticus and amygdala kindling rat models [36,37,38]. Short electrical stimulation trains can reduce the pathological electrophysiological modifications observed in epilepsy [39,40].

Despite its proven effectiveness, very little is known about the changes caused by DBS at the cellular level [41,42]. Experimental models can help study neural lesions and evaluate and improve electrical stimulation efficacy [43]. A single stimulation train increased c-Fos expression in the amygdala and other limbic areas, but not in the hippocampus of healthy rats [44]. In contrast, amygdala low-frequency stimulation (LFS) with 4 Hz restored altered glutamatergic and GABAergic gene expressions in CA1 pyramidal neurons [45]. Furthermore, selective activation of GABAergic hippocampal cells with optical LFS is effectively reducing epileptiform activity [46]. DBS of the thalamus had antiapoptotic and anti-inflammatory effects and increased adenosine levels in the hippocampus of pilocarpine-treated rats [47,48]. Moreover, it inhibited the abnormal mossy fiber sprouting in the kainic acid (KA) model [49]. Anterior thalamic nuclei stimulation of pilocarpine treated rats suppressed neurogenesis in the hippocampus 28 days after SE, but this effect disappeared after 60 days [50].

We previously reported that 4 Hz stimulation of the amygdala for 4 × 50 s for 10 days had a marked effect on the electrophysiological activity of the hippocampus, increasing the theta power and reducing the pathological phase-amplitude coupling in the pilocarpine model of epilepsy [39]. The present study aimed to extend these observations and determine whether amygdala low-frequency stimulation has a disease-modifying effect by preventing some of the previously observed modifications of interneuron density caused by epileptogenesis. By this, we aim to gain a deeper insight into the mechanisms by which DBS can exert its anticonvulsant effect.

## 2. Materials and Methods

### 2.1. Animals

All experiments were carried out according to the 2010/63/EU directive of the European Parliament and approved by the Ethics Committee for Scientific Research of the George Emil Palade University of Medicine, Pharmacy, Science, and Technology of Târgu Mureș (ethical committee license nr: 340/17 November 2017 extended by nr. 54/2 April 2019). For the experiments, 6-weeks old male Wistar rats were used from the aforementioned university’s breeding facility. They were kept under standard environmental and light conditions (12/12 h dark/light cycle, light on at 7 a.m.) with *ad libitum* access to food and water.

### 2.2. Lithium-Pilocarpine Model

Epilepsy was induced using the lithium-pilocarpine protocol of temporal lobe epilepsy (LiCl: 3 mEq/kg i.p., 16 h before pilocarpine-hydrochloride, 30 mg/kg, i.p.,), which caused status epilepticus (SE) with generalized tonic-clonic seizures. Lithium was given to potentiate the effect of pilocarpine, while methylscopolamine (1 mg/kg) was given 20 min before pilocarpine administration to reduce the undesirable peripheral cholinergic effects caused by pilocarpine. All substances used for epilepsy induction were purchased from Sigma-Aldrich (St. Luis, MO, USA). Rats were continuously video monitored and observed by experimenters after pilocarpine was injected. Seizures were classified by three experienced researchers using the revised Racine scale (R) [51]. Only rats exhibiting Racine 5- or 6-grade seizures were included in the study.

After 2 h of SE, diazepam (5 mg/kg, i.p., Terapia-Ranbaxy, Cluj-Napoca, Romania) was injected to stop the status and repeated during the following 24 h if convulsive behaviors persisted or reappeared. Animals were rehydrated, and their diet was supplemented to ensure survival. Control animals were kept in similar housing conditions and diet as pilocarpine-treated rats. They received only lithium and i.p. saline solution and a single diazepam dose 2 h after saline administration. After SE induction, animals were placed in individual cages and video-monitored throughout the study’s whole duration (approx. 3 months).

In total, 32 animals were used for the study, out of which 20 received pilocarpine (Pilo). All of the Pilo animals exhibited status epilepticus. Two out of 20 animals had only R1–R2 grade seizures during SE and were excluded from the study. The rest of the animals (18) had at least 1 Racine 5 seizure, all of them survived the SE and the first 24 h, but 4 of them died on the following days despite our comprehensive care.

Pilocarpine group: animals were randomly assigned to 2 subgroups: the deep brain stimulation group (DBS-Pilo) and the sham-operated group (SHAM-Pilo). DBS-Pilo (*n* = 10) animals were implanted with stimulation and recording electrodes and underwent the stimulation protocol (see below), while SHAM-Pilo animals (*n* = 4) were implanted with electrodes but were not stimulated. One SHAM-Pilo animal had unsuccessful tissue fixation, and thereby it was excluded from the study.

Control group: 12 age-matched control Wistar rats were used for the study. Control animals (*n* = 8) consisted of 3 animals implanted with electrodes but not stimulated, and 5 animals were not implanted with electrodes. DBS-control animals (*n* = 4) underwent the same stimulation protocol as the DBS-Pilo group. One animal from the DBS-control group had unsuccessful transcardial perfusion. Consequently, its histological data were not included in the study.

### 2.3. Electrode Implantation Protocol

During the surgical procedure, all efforts were made to minimize pain and suffering. Pilo rats (approx. 8 weeks after SE) and the control animals underwent stereotaxic electrode implantation protocol. Anesthesia was induced by isoflurane (5%) followed by intramuscular injection of a mixture of ketamine (100 mg/kg LeVet. Beheer B.V., Oudewater, Netherlands) and Xylazine (10 mg/kg, Bioveta, Ivanovice na Hané, Czech Republic) cocktail. The electrode implantation protocol used bregma as a reference and was performed according to the Paxinos and Watson’s atlas for rats [52]. The stimulation electrode, a bipolar polyimide coated twisted stainless-steel electrode (diameter: 0.2 mm, tip distance: 0.125 mm), was implanted in the left basolateral amygdala (BLA) (AP − 2.8 mm; ML + 5 mm to bregma; DV + 8.4 mm to skull surface). Two recording electrodes were implanted bilaterally in the hippocampus (AP: −3.6 mm, ML: ±2.6 mm, DV: 3.6 mm). Two stainless-steel screw electrodes were used as epidural recording electrodes (AP: +2 mm, ML: ±2 mm). The other two were placed at the posterolateral surface of the parietal bones and used as reference and ground. The electrodes were connected to two connectors fixed to the skull with dental acrylic (Duracryl, Spofa Dental, Czech Republic). All electrodes were purchased from Plastics One Inc. (Roanoke, VA, USA). We used local anti-inflammatory and antibiotic solutions (Tobramycin-Dexamethasone) at the end of the surgery. Correct electrode positioning was verified by Nissl staining at the end of the experiments.

### 2.4. Stimulation Protocol

After a 10-day post-surgical recovery, animals were placed in a custom-built Plexiglas cage (40 × 45 × 50 cm^3^) housed in a Faraday cage in standard conditions. The connectors were attached to a swivel contact (SL6X2C, Plastics One Inc., Roanoke, VA, USA), allowing stimulation and recording EEG signals simultaneously on a freely moving rat. Stimulation was done using the BioStim gate-controller, and pulse pattern generator (STC-8b) connected to a bipolar floating end-stage (BSE-3b; both from Supertech, Pécs, Hungary). The stimulation signal parameters were verified at the beginning and end of the study by a shunt resistor connected to an oscilloscope.

Biphasic square pulses (duration: 100 µs, amplitude: 500 µA) were used at 4 Hz (regular interpulse interval). The animals received a daily package of 4 × 50 s of training with a 5 min pause between each training for 10 days. The stimulation was performed in the same period of the day each time (between 3 p.m. and 4 p.m.). EEG was recorded starting 5 min before the first stimulus train and stopped 5 min after the last. During these 10 days, continuous video recording was maintained and analyzed as previously described.

### 2.5. Histology and Immunocytochemistry

At the end of the stimulation protocol, rats were deeply anesthetized with a ketamine-xylazine mixture (90 mg/kg + 10 mg/kg) and transcardially perfused with ice-cold normal saline solution (0.9%) for 1 min. This was followed by 4% paraformaldehyde in 0.1 M phosphate buffer (pH 7.4) solution containing 15% picric acid, with a 20 mL/min perfusion rate for 20 min. The brains were postfixed for 24 h in 4% paraformaldehyde.

Then, 60 µm thick coronal sections were cut along the septotemporal axis containing the whole dorsal hippocampus with a vibratome (Leica, VT 1000S, Nussloch, Germany). Correct electrode positions were tracked by using Nissl-staining followed by light microscopy.

For immunocytochemical labeling, we used sections starting from the anterior part of the hippocampus, where all subfields could already be observed (approx. AP −3.14 to bregma). One to three more sections were analyzed in every animal, each at 180 µm posterior from the previous along the A-P axis. Sections were washed 3 times for 10 min in 0.1 M PB, then in Tris-buffered saline (pH 7.4) containing 0.1% Triton-X (TBS-T), followed by incubation in a solution containing 10% normal horse serum (NHS; Vector Laboratories, Burlingame, CA, USA) in TBS-T for 50 min to block nonspecific binding of antibodies.

Primary antibodies against parvalbumin (PV; polyclonal-guinea pig raised, dilution 1:2000; Synaptics Systems product no: 195004), neuronal nitric oxide synthase (nNOS; mouse raised-monoclonal nNOS-B1-IgG1 isotype-Sigma-N2280, dilution 1:500), and neuropeptide Y (NPY; rabbit raised monoclonal; Immuno star: 22940, dilution 1:500) were applied in 0.1% TBS-T with 1% NHS and incubated overnight at room temperature. After a 3 × 10 min wash in TBS-T, sections were incubated for 4 h using a mixture of the appropriate fluorophore-conjugated secondary antibodies applied in 0.1% TBS-T with 1% NHS at room temperature. Secondary antibodies were the following: donkey raised anti guinea pig Alexa Fluor 488 (dilution: 1:500, Jackson Immuno Research, product code 706-545-148), donkey raised anti-mouse Alexa 647 (dilution 1:500, Jackson Immuno Research, product code 715-605-151), donkey raised anti-rabbit indocarbocyanine 3 (CY3, dilution 1:500, Jackson Immuno Research, product code 711-165-152). After incubation, sections were washed 3 times in TBS, mounted on glass slides, coverslipped with Vectashield (Vector Laboratories, Burlingame, CA, USA), and isolated with nail varnish.

To test the nonspecific binding of secondary antibodies, sections were prepared with the same protocol but without primary antibodies. Images were acquired with the same parameters, and no cell-specific signal similar to the full reaction was observed. Cross-reactivity of the secondary antibodies was tested by applying all three secondary antibodies simultaneously with one primary at a time. No labeling was observed after combing the unrelated secondary antibodies. Tissue autofluorescence was low, and it was not affecting the signal detection.

Sections were scanned by a Leica SP8 TCS confocal system with an inverted 20× dry objective. The complete hippocampus was scanned from each section by creating tile- and z-stack images. Tile-stacks contained 6 × 3 to 7 × 4 scanning fields (depending on the hippocampal surface area). Z-stacks consisted of 9–10 layers taken at 5 µm intervals. The image of the whole hippocampal surface was acquired in three different channels. Hippocampal CA1 layer borders (oriens, pyramidale, radiatum, and lacunosum-moleculare) were marked, and their area was measured using the Fiji software (Extension to Image-J, NIH, USA). The volume was calculated by multiplying the area by the Z-stack thickness. Cells were counted manually using the Fiji software’s cell counter function, then reported as cell number/volume, thereby determining the cell density of different cell types. The average number of different neuron types in a given region was obtained from bilateral (left and right hippocampal) counts.

Hippocampal sclerosis frequently occurs in TLE, and previous studies from our group had reported hippocampal shrinkage in chronic epileptic rats [24]. If the cell number is constant, the cellular density apparently can increase due to the shrinkage. In order to offset the effect of tissue shrinkage, a correction factor was introduced. A 3D correction factor was calculated from the ratio of the mean CA1 surface of the pilocarpine treated rats and control rats. All densities presented in this study are corrected densities.

### 2.6. Statistics

Normality was checked using the Shapiro–Wilk, and D’Agostino and Pearson’s test, and parametric statistical group comparisons were made by using one-way ANOVA for equal variances and Welch’s ANOVA for unequal variances with the appropriate post hoc test for multiple comparisons. Data were represented as mean±SEM, and the alpha value was set to 0.05 if not stated otherwise.

All statistical analyses and graphs were done with GraphPad Prism version 8.

## 3. Results

All rats from the pilocarpine group had spontaneous recurrent seizures based on the video recordings. The control animals had no seizures at all. There was a significant reduction in seizure duration in the DBS group and a reduction in seizure rate. Electrode implantation did not affect seizure rate or seizure duration.

The area of the CA1 hippocampal subfield was significantly decreased in the pilocarpine group by 36.6% (*p <* 0.0001).

**Figure 1:**
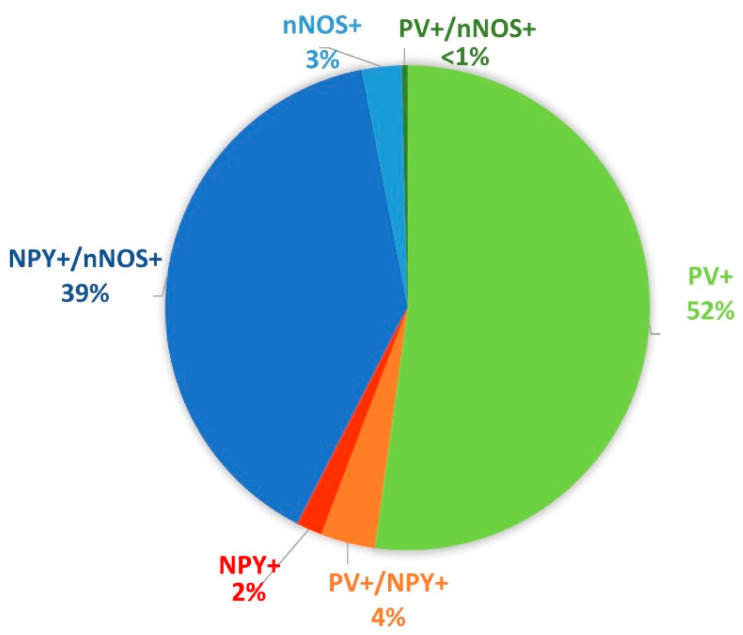
Proportional distribution of the different cell types based on the protein expression profile in the CA1 area of the control animals (including also the lacunosum moleculare layer). The largest populations were the parvalbumin (PV)+ cells (green) and neuropeptide Y (NPY)+/ neuronal nitric oxide synthase (nNOS)+ cells (blue).

We found single labeled cells (only PV+ or NPY+ or nNOS+) and all possible combinations of double labeling. We did not find any triple labeled cells. In the CA1 area of the control animals (including also the lacunosum moleculare layer), the most abundant cell types were the PV+ cells (1394 ± 117.3 cells/mm^3^) and the NPY+/nNOS+ cells (1054.54 ± 91.9 cells/mm^3^). The rest of the cell types (NPY+, nNOS+, PV+/NPY+, PV+/nNOS+) were much rarer (Figure 1).

### 3.1. PV+ Cells

**Figure 2:**
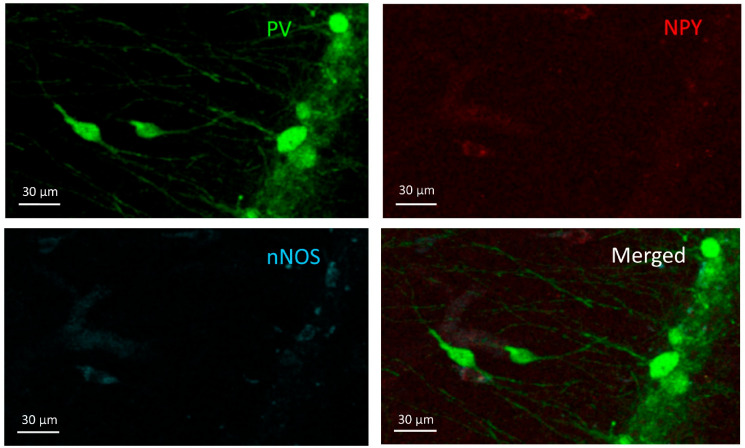
PV+ cells. Two PV+ cells can be seen in the stratum radiatum (**middle-left**) and more PV+ cells in the stratum pyramidale (**right**) on the PV channel image (**green**) but do not appear in the other two channels (NPY-red and nNOS-blue).

**Figure 3:**
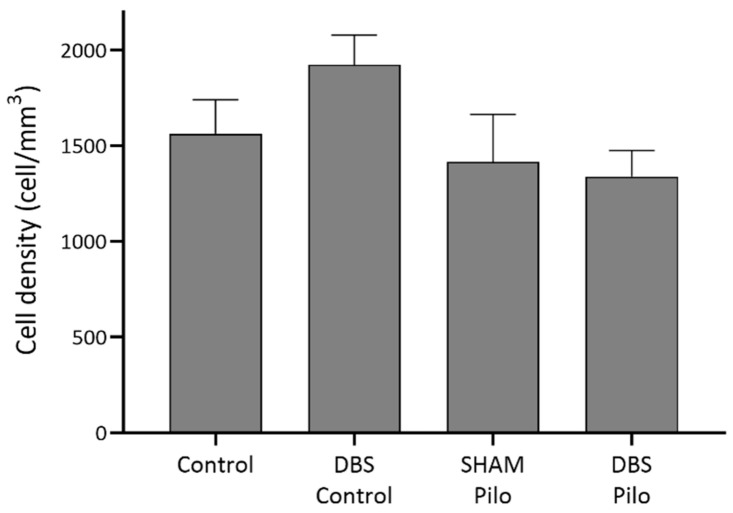
PV+ cell density in stratum oriens, pyramidale, and radiatum of the CA1 subregion of the hippocampus. Deep brain stimulation (DBS) was applied to the basolateral amygdala for 10 days in control (DBS-control) and pilocarpine-induced animals (DBS-Pilo) and compared to controls (control, *n* = 8) and pilocarpine-induced animals (SHAM-Pilo). No significant differences were observed among the groups. Data are presented as means ± SEM.

Cells in this group expressed parvalbumin (PV+) but were negative for NPY and nNOS (Figure 2). They were found in stratum oriens, pyramidale, and radiatum, but their majority was in the stratum oriens and pyramidale. PV+ cell density did not show significant differences among the groups (F(3,20) = 1.441, *p* = 0.26) (Figure 3).

### 3.2. PV+/NPY+ Cells

**Figure 4:**
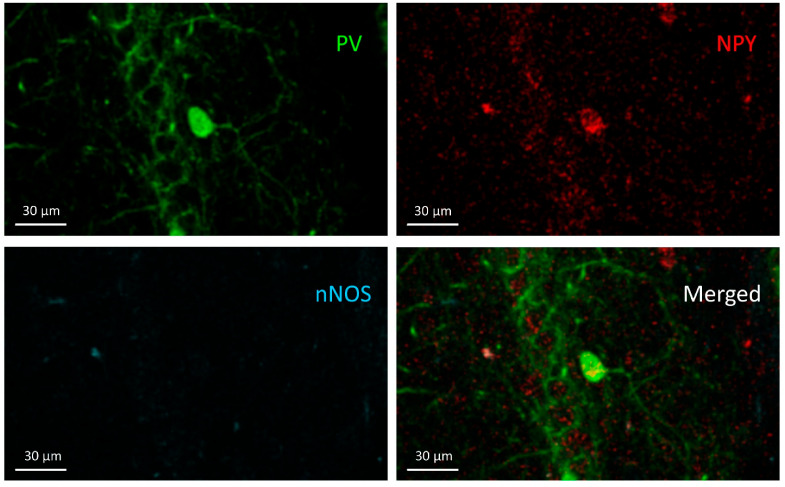
PV+/NPY+ cell; the cell can be seen in the PV (green) and NPY (red) channels in the stratum pyramidale but does not appear in the nNOS channel.

**Figure 5:**
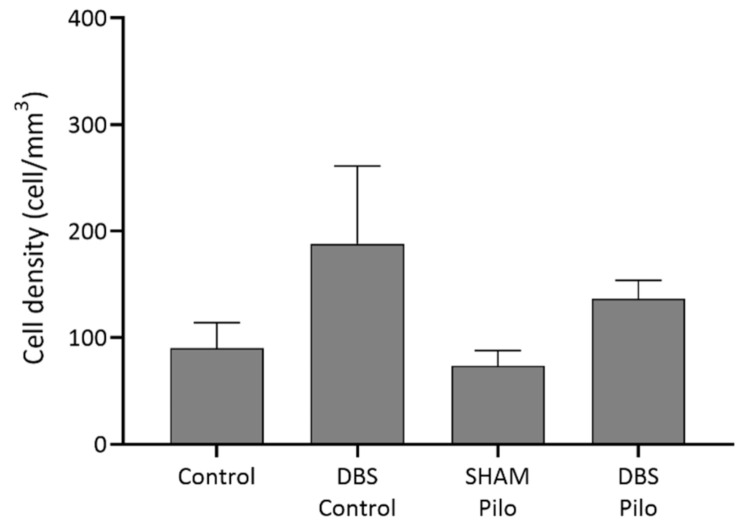
PV+/NPY+ cell density in the CA1 subregion of the hippocampus. No significant differences were observed among the groups. The density was higher in the DBS groups, but the difference was not significant, and the density of this cell type was very low compared to other cell types. Data are presented as means ± SEM.

Cells co-expressing PV and NPY are bistratified cells. These cells are negative for nNOS (Figure 4). Regarding PV+/NPY+ cell numbers, no significant overall differences were observed between the groups (F(3,20) = 2.173, *p* = 0.1229) (Figure 5).

### 3.3. NPY+ Cells

**Figure 6:**
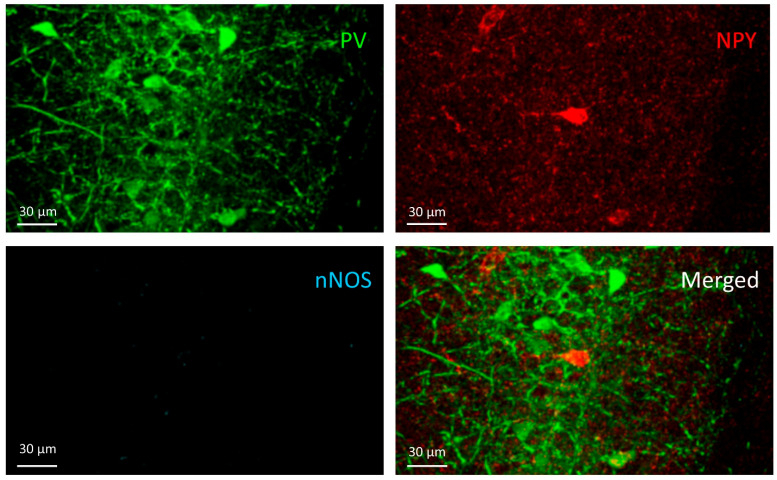
The NPY+ cell can be seen on the NPY channel image (**red**) in the pyramidal layer of CA1 but does not appear in the other two channels (PV and nNOS).

**Figure 7:**
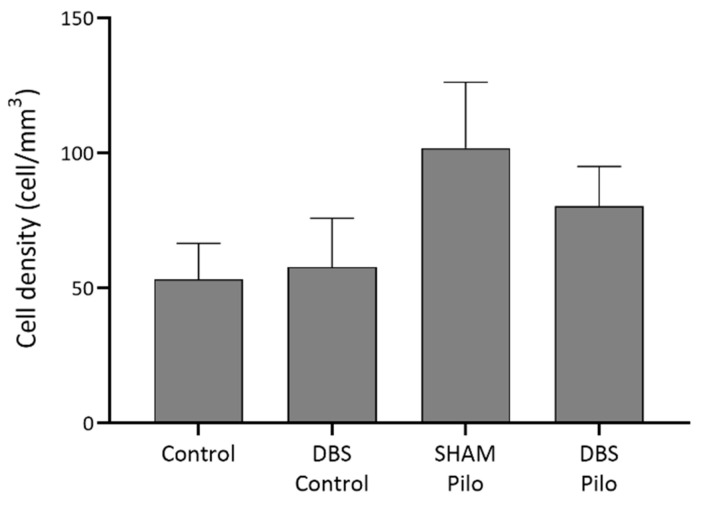
NPY+ cell density in the CA1 region of the hippocampus. No significant differences were observed among the groups, despite that Pilo animals had higher densities. Data are presented as means ± SEM.

The NPY+ (PV– and nNOS–) cells (Figure 6) were much rarer than PV+ cells in our data. There was no significant difference in cell density among groups (F(3,20) = 1.3, *p* = 0.299) (Figure 7), despite that Pilo animals had higher densities.

### 3.4. NPY+/nNOS+ Cells

**Figure 8:**
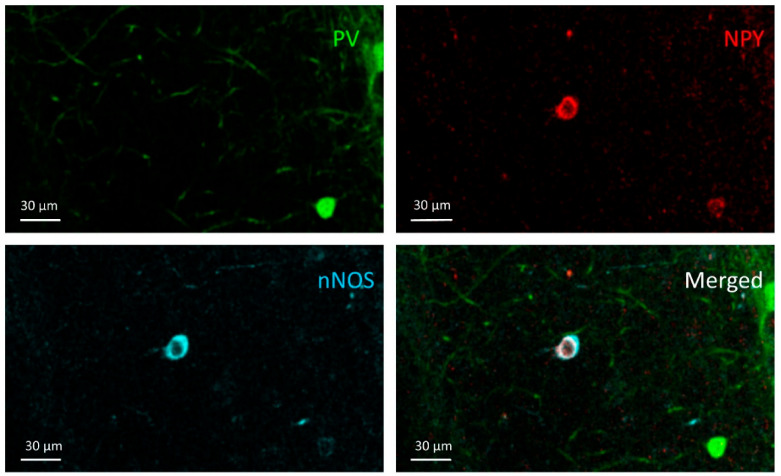
NPY+/nNOS+ cell; An NPY+/nNOS+ cell can be seen in the NPY and nNOS channel images in the stratum radiatum of the CA1, but it does not appear in the PV channel.

**Figure 9:**
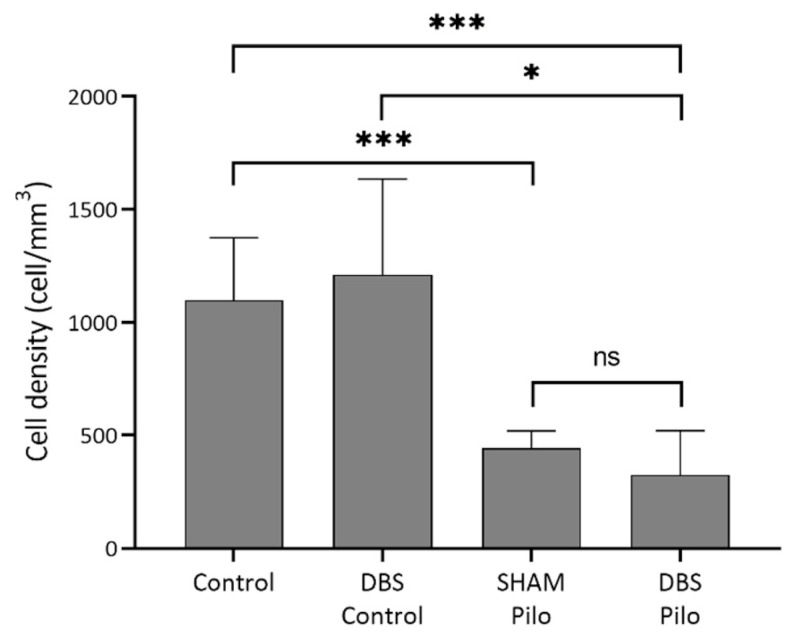
NPY+/nNOS+ cell density in the CA1 subregion of the hippocampus. There was a significant overall difference among groups (Welch’s ANOVA test). Multiple comparisons have shown that mean cell density was significantly lower in pilocarpine animals compared to controls. At the same time, there was no significant difference between the sham-operated group (SHAM-Pilo) and deep brain stimulation group (DBS-Pilo) groups. Data are presented as means ± SEM. * p < 0.05, *** p < 0.001, ns: no significant difference.

Cells simultaneously expressing NPY and nNOS are ivy cells in the stratum oriens, pyramidale, and radiatum (Figure 8). There was significant overall difference among NPY+/nNOS+ cell density (*W* = 14 (3.0, 6.97); *p* = 0.002, Welch’s ANOVA test). The mean cell density was the lowest in the DBS-Pilo group and highest in the DBS-control group. Multiple comparisons (Benjamini and Hochberg posttest) had shown that mean cell density was significantly lower in pilocarpine-treated animals (SHAM-Pilo vs. control 463.5 ± 27.9 vs. 1139 ± 108.9; *p <* 0.001 and DBS-Pilo vs. DBS-control 391.8 ± 91.68 vs. 1260 ± 202.7; *p <* 0.0.05 (mean ± SEM)). There was no significant difference between the SHAM-Pilo and DBS-Pilo groups (Figure 9).

## 4. Discussion

To test whether DBS influences interneuron cell density in epileptic rats, we evaluated the density of different types of interneurons, including the two major subclasses of interneurons (PV expressing interneurons and NPY/nNOS expressing cells). Our results show a sparing of PV+ and PV+/NPY+ cell density in the CA1 region of the hippocampus of pilocarpine treated rats, and the amygdala low-frequency stimulation did not influence these densities. NPY+/nNOS+ cell density was significantly decreased in the epileptic group, and LFS could not influence this decrease.

Changes in cell numbers in the hippocampus, especially that of interneurons, occur in TLE. Still, there is insufficient data regarding the possible effect of antiepileptic treatment on these changes [6], and treatments that could prevent or diminish pathological cell loss and stop the progressive feature of TLE are lacking [53,54]. Deep brain stimulation is a novel, promising antiepileptic therapeutic tool in rodents and humans [55,56].

*PV+ cell density:* We found that PV+ cell density was not modified by the epileptogenic processes nor by DBS. According to literature PV+ cells (mainly basket + axo-axonic + O-LM) represent 31% of all interneurons in CA1 area [19,20]. They are fast-spiking inhibitory cells with a role in generating pyramidal cell oscillations. Increased or decreased activation of these cells can contribute to hypersynchrony or hyperexcitability of hippocampal activity, subsequently leading to seizures [11]. Bistratified cells are also PV+ fast-spiking inhibitory neurons. They are targeting pyramidal cell’s dendritic domains and provide pathway-specific inhibition by controlling the excitatory inputs to pyramidal cells [21]. PV+/NPY+ (bistratified) cells make up around 6% of all hippocampal cells, but not all bistratified cells express NPY [11]. In our study, their proportion was 4%. Compared to the PV+ cells, their ratio is very low, thereby their role in the altered, epileptogenic network activity may be less important than that of PV+s, and the increased density in the DBS groups (which was not significant) may be less interpretable.

The changes in the density of PV+ cells during epileptogenesis are not unequivocal. It was reported that PV+ cell density was reduced in the CA1 region during the first two weeks following pilocarpine-induced SE [22]. Likewise, André et al. reported that this decrease could be observed in the stratum oriens already 24 h after Li-pilocarpine-induced SE and persisted after 2–3 weeks, while no loss of PV+ cells occurred in the stratum radiatum and pyramidale [57]. Intrahippocampal injection of KA caused a loss of PV+ and NPY+ cells at injection sites 2 days after SE, the loss being more pronounced after 21 days [58]. When examined two weeks after kainate-induced SE, a similar decrease in PV+ cell numbers was observed [59].

Contrarily, Long et al. observed that during the first week following the pilocarpine-induced SE, PV+ cell density was preserved in the CA1 region, while after two weeks, their numbers were already higher. This increase was present even 60 days after SE [60]. Previous findings in our laboratory had shown similar results: PV expressing cell densities were preserved in the pilocarpine model 4–5 months after SE, so at a later time stage than in the present study [24]. Preservation of PV density could indicate a possible resistance of PV cells to cell death [61]. Moreover, their increased perisomatic bouton numbers on pyramidal cells [60,62] could suggest that perisomatic inhibition is intensified in epileptic animals in a given period of the disease, leading to pathological hyper-synchronism [63]. In chronic hippocampal epileptic tissue, removed during epilepsy surgery, decreased PV+ cell density was reported, despite the sparing of perisomatic GABAergic innervation [23,64].

Preservation of the PV+/NPY+ bistratified cell numbers was already reported in the pilocarpine model in the early latent phase [22]. Our results extend that observations, showing preserved cell densities in the early chronic stage of the disease.

*Effect of DBS on PV+ cell density*: pilocarpine-treated rats that underwent DBS had slightly lower cell density than non-stimulated rats, but this difference was not significant. If the preservation of the PV cell density of PV expression in a shrunken hippocampal volume is a driving mechanism of epileptogenesis, this short DBS protocol does not alleviate it. As the stimulation did not influence the PV cell density in healthy rats, we consider that DBS does not impact the PV cell density. The possible therapeutic effect is not expressed through the PV cells if the DBS is applied in the short-term.

*NPY+/nNOS+ cell density*: In our study, there was a significant decrease in NPY+/nNOS+ cell density in epileptic animals, and this was not modified by DBS.

NPY+/nNOS+ cells are a group of dendrite innervating GABAergic neurons, and they represent an abundant type of interneurons in the CA1 [27]. They are ivy cells in the stratum oriens and pyramidale and neurogliaform cells in stratum L-M of the CA1 area. In the stratum radiatum, around 80% are ivy, and 20% are neurogliaform cells, respectively [20,26]. NPY+/nNOS+ cells (ivy and neurogliaform cells) represent around 37% of all CA1 inhibitory cells [19,65]. They regulate excitability through slow GABAergic inhibition, but they may also play a role in maintaining oscillations by fast inhibition [65,66]. Moreover, by synthesizing NO and NPY, they modulate general excitability and synaptic plasticity and play an important role in the epileptogenic processes [25,28]. Selective blockage of nNOS can shorten seizure duration and severity [67]. In the cerebral cortex of epileptic patients, nNOS expression is increased [68]. However, the relationship of NO with epileptogenesis is controversial, as others found that lamotrigine has anticonvulsant action partially by reducing the nitric oxide levels in the brain [69]. Moreover, NO increased the proliferation of neural stem cells and maintained long-term neuroinflammation in KA-treated mice’s hippocampus [70].

nNOS+ cell numbers were preserved two weeks after SE in the hippocampus of pilocarpine rats, but 10 months after SE, their numbers were decreased significantly [71]. Like this, in advanced stages of epilepsy, nNOS+ cell numbers were decreased in the human hippocampus [28].

It is noteworthy that those studies did not use NPY to detect colocalization of NPY and nNOS, while in this study, both NPY and nNOS labeling were used. Our laboratory’s previous work had shown that 4–5 months after pilocarpine-induced SE, the nNOS+/NPY+ cell density was significantly decreased [24]. The present work demonstrates that the same alteration of cell density is already present three months after SE in Li-Pilocarpine rats. Park et al. found that following kainate-induced SE, the number of NPY+/nNOS+ neurons in the CA1 region of the hippocampus was already decreased after the first 20 days [72].

Studies that used the only NPY as a marker reported that the loss of NPY+ cells in the hippocampus’s hilus was present already 5–10 days after kainate and lithium-pilocarpine-induced SE [60,73]. At the same time, in contrast with our observations, in the CA1 and CA3 subfields, the NPY expressing cell density remained unchanged compared to controls during the first two months after SE [58,60]. This means that the reduction of cell density may take place between the second and fourth months after SE.

Cells that express NPY and are negative for PV and nNOS are probably from the group of cells named double-projection cells, and their density is very low compared to the other groups [18,74]. In our study, in the epileptic animals, only NPY+ cell density was increased but not significantly compared to controls. A study reported that only NPY+ cell density was increased in the hippocampal CA1 region of rats 10 days after kainic acid injection [72]. As the previously mentioned studies using NPY did not use nNOS labeling, our results are not comparable, as most NPY+ cells in the hippocampus are nNOS+ too [58,60,73,75]. We found a few very large NPY+/nNOS+ cells around stratum pyramidale, intensely stained with nNOS+, which represent a rare type of projection neurons [10,26]. They were not taken into account during statistics.

*Effect of DBS on NPY+/nNOS+ cell density*: To our knowledge, there is no data on the impact of low-frequency electrical stimulation on hippocampal NPY+/nNOS+ interneuron density. In our study, DBS did not affect these cell numbers in controls or the epileptic group. However, these findings should be interpreted carefully due to the relatively low sample size of the DBS-control and SHAM-Pilo groups.

As PV, NPY, and nNOS are activity-dependent proteins [26,73,76], it may be that some of the cells were not expressing these proteins in a detectable amount all time, thereby the real cell numbers could be higher in all groups, even if Wang et al. found that the percentage of PV+, NPY+, and nNOS+ cells expressing c-Fos was significantly higher in pilocarpine treated rats 10 weeks after SE [77]. The decreased NPY/nNOS cell density may be partially explained by changes in detectability of the proteins or reduced or disappeared expression [60].

## 5. Conclusions

PV expressing cell density was preserved in the CA1 area of the hippocampus of pilocarpine treated rats, while the NPY/nNOS expressing cell density was significantly decreased three months after SE. Decreased density of ivy cells may facilitate increased general excitability and reduce the seizure threshold. In contrast, the persistence of PV+ cell numbers in a shrunken hippocampus might facilitate local hypersynchrony and promote epileptogenesis. Low-frequency electrical stimulation of the left basolateral amygdala did not influence the PV+ cell density and the decrease of the NPY+/nNOS+ cell density. Low-frequency stimulation has a robust effect in the short term on seizure rates and pathological electrophysiological activity; however, our study suggests that this effect does not occur as a result of modified interneuron densities. Nevertheless, further studies should address the effect of stimulation protocols applied for longer periods on the morphological reorganization of the hippocampus.

## Data Availability

The datasets generated and/or analyzed in this study are available on reasonable request.

## Abbreviations

BLA	Basolateral amygdala
CA1	Cornu ammonis 1
DBS	Deep brain stimulation
EEG	Electroencephalography
GABA	Gamma-aminobutyric acid
HFS	High-frequency stimulation
LFS	Low-frequency stimulation
NHS	Normal horse serum
nNOS	Neuronal nitric oxide synthase
NPY	Neuropeptide y
O-LM	Oriens-lacunoscum moleculare
PB	Phosphate buffer
Pilo	Pilocarpine
PTZ	Pentylenetetrazole
PV	Parvalbumin
SE	Status epilepticus
SEM	Standard error of the mean
SRSs	Spontaneous recurrent seizures
TBS	Tris-buffered saline
TLE	Temporal lobe epilepsy

## References

[1] Sander J.W., Shorvon S.D. (1996). Epidemiology of the Epilepsies. J. Neurol. Neurosurg. Psychiatry.

[2] Wilner A.N., Sharma B.K., Soucy A., Krueger A. (2012). Health Plan Paid Cost of Epilepsy in 2009 in the U.S. Epilepsy Behav..

[3] Wiebe S. (2000). Epidemiology of Temporal Lobe Epilepsy. Can. J. Neurol. Sci..

[4] Berg A.T., Berkovic S.F., Brodie M.J., Buchhalter J., Cross J.H., Van Emde Boas W., Engel J., French J., Glauser T.A., Mathern G.W. (2010). Revised Terminology and Concepts for Organization of Seizures and Epilepsies: Report of the ILAE Commission on Classification and Terminology, 2005–2009. Epilepsia.

[5] Mohanraj R., Brodie M.J. (2006). Diagnosing Refractory Epilepsy: Response to Sequential Treatment Schedules. Eur. J. Neurol..

[6] Thom M. (2009). Hippocampal Sclerosis: Progress since Sommer. Brain Pathol..

[7] Buckmaster P.S. (2010). Mossy Fiber Sprouting in the Dentate Gyrus. Epilepsia.

[8] Zhang S., Khanna S., Tang F.R. (2009). Patterns of Hippocampal Neuronal Loss and Axon Reorganization of the Dentate Gyrus in the Mouse Pilocarpine Model of Temporal Lobe Epilepsy. J. Neurosci. Res..

[9] Botterill J.J., Brymer K.J., Caruncho H.J., Kalynchuk L.E. (2015). Aberrant Hippocampal Neurogenesis after Limbic Kindling: Relationship to BDNF and Hippocampal-Dependent Memory. Epilepsy Behav..

[10] Freund T.F., Buzsaki G. (1996). Interneurons of the Hippocampus. Hippocampus.

[11] Somogyi P., Klausberger T. (2005). Defined Types of Cortical Interneurone Structure Space and Spike Timing in the Hippocampus. J. Physiol..

[12] Somogyi P. (2010). Hippocampus: Intrinsic Organization. Handbook of Brain Microcircuits.

[13] Liu Y.Q., Yu F., Liu W.H., He X.H., Peng B.W. (2014). Dysfunction of Hippocampal Interneurons in Epilepsy. Neurosci. Bull..

[14] Maglóczky Z., Freund T.F. (2005). Impaired and Repaired Inhibitory Circuits in the Epileptic Human Hippocampus. Trends Neurosci..

[15] Sloviter R.S. (1991). Permanently Altered Hippocampal Structure, Excitability, and Inhibition after Experimental Status Epilepticus in the Rat: The “Dormant Basket Cell” Hypothesis and Its Possible Relevance to Temporal Lobe Epilepsy. Hippocampus.

[16] Szilagyi T., Szava I., Metz E.J., Mihaly I., Orban-Kis K. (2014). Untangling the Pathomechanisms of Temporal Lobe Epilepsy-The Promise of Epileptic Biomarkers and Novel Therapeutic Approaches. Brain Res. Bull..

[17] Gáll Z., Kelemen K., Mihály I., Salamon P., Miklóssy I., Zsigmond B., Kolcsár M. (2020). Role of Lacosamide in Preventing Pentylenetetrazole Kindling-Induced Alterations in the Expression of the Gamma-2 Subunit of the GABAA Receptor in Rats. Curr. Mol. Pharmacol..

[18] Harris K.D., Hochgerner H., Skene N.G., Magno L., Katona L., Bengtsson Gonzales C., Somogyi P., Kessaris N., Linnarsson S., Hjerling-Leffler J. (2018). Classes and Continua of Hippocampal CA1 Inhibitory Neurons Revealed by Single-Cell Transcriptomics. PLoS Biol..

[19] Pelkey K.A., Chittajallu R., Craig M.T., Tricoire L., Wester J.C., McBain C.J. (2017). Hippocampal Gabaergic Inhibitory Interneurons. Physiol. Rev..

[20] Bezaire M.J., Soltesz I. (2013). Quantitative Assessment of CA1 Local Circuits: Knowledge Base for Interneuron-Pyramidal Cell Connectivity. Hippocampus.

[21] Buhl E.H., Szilágyi T., Halasy K., Somogyi P. (1996). Physiological Properties of Anatomically Identified Basket and Bistratified Cells in the CA1 Area of the Rat Hippocampus in Vitro. Hippocampus.

[22] Dinocourt C., Petanjek Z., Freund T.F., Ben-Ari Y., Esclapez M. (2003). Loss of Interneurons Innervating Pyramidal Cell Dendrites and Axon Initial Segments in the CA1 Region of the Hippocampus Following Pilocarpine-Induced Seizures. J. Comp. Neurol..

[23] Andrioli A., Alonso-Nanclares L., Arellano J.I., DeFelipe J. (2007). Quantitative Analysis of Parvalbumin-Immunoreactive Cells in the Human Epileptic Hippocampus. Neuroscience.

[24] Orbán-Kis K., Szabadi T., Szilágyi T. (2015). The Loss of Ivy Cells and the Hippocampal Input Modulatory O-LM Cells Contribute to the Emergence of Hyperexcitability in the Hippocampus. Rom. J. Morphol. Embryol..

[25] Colmers W.F., El Bahh B. (2003). Neuropeptide Y and Epilepsy. Epilepsy Curr..

[26] Somogyi J., Szabo A., Somogyi P., Lamsa K. (2012). Molecular Analysis of Ivy Cells of the Hippocampal CA1 Stratum Radiatum Using Spectral Identification of Immunofluorophores. Front. Neural Circuits.

[27] Armstrong C., Krook-Magnuson E., Soltesz I. (2012). Neurogliaform and Ivy Cells: A Major Family of NNOS Expressing GABAergic Neurons. Front. Neural Circuits.

[28] Leite J.P., Chimelli L., Terra-Bustamante V.C., Costa E.T., Assirati J.A., de Nucci G., Martins A.R. (2002). Loss and Sprouting of Nitric Oxide Synthase Neurons in the Human Epileptic Hippocampus. Epilepsia.

[29] Wyeth M., Nagendran M., Buckmaster P.S. (2020). Ictal Onset Sites and γ-Aminobutyric Acidergic Neuron Loss in Epileptic Pilocarpine-Treated Rats. Epilepsia.

[30] Engel J. (1996). Surgery for Seizures. N. Engl. J. Med..

[31] Wiebe S., Blume W.T., Girvin J.P., Eliasziw M. (2001). A Randomized, Controlled Trial of Surgery for Temporal-Lobe Epilepsy. N. Engl. J. Med..

[32] Velasco A.L., Velasco M., Velasco F., Menes D., Gordon F., Rocha L., Briones M., Márquez I. (2000). Subacute and Chronic Electrical Stimulation of the Hippocampus on Intractable Temporal Lobe Seizures: Preliminary Report. Arch. Med. Res..

[33] Velasco A.L., Velasco F., Velasco M., Trejo D., Castro G., Carrillo-Ruiz J.D. (2007). Electrical Stimulation of the Hippocampal Epileptic Foci for Seizure Control: A Double-Blind, Long-Term Follow-up Study. Epilepsia.

[34] Cukiert A., Cukiert C.M., Burattini J.A., Mariani P.P., Bezerra D.F. (2017). Seizure Outcome after Hippocampal Deep Brain Stimulation in Patients with Refractory Temporal Lobe Epilepsy: A Prospective, Controlled, Randomized, Double-Blind Study. Epilepsia.

[35] Tyrand R., Seeck M., Spinelli L., Pralong E., Vulliémoz S., Foletti G., Rossetti A.O., Allali G., Lantz G., Pollo C. (2012). Effects of Amygdala-Hippocampal Stimulation on Interictal Epileptic Discharges. Epilepsy Res..

[36] Asgari A., Semnanian S., Atapour N., Shojaei A., Moradi H., Mirnajafi-Zadeh J. (2014). Combined Sub-Threshold Dosages of Phenobarbital and Low-Frequency Stimulation Effectively Reduce Seizures in Amygdala-Kindled Rats. Neurol. Sci..

[37] Zhong K., Wu D.C., Jin M.M., Xu Z.H., Wang Y., Hou W.W., Li X.M., Zhang S.H., Chen Z. (2012). Wide Therapeutic Time-Window of Low-Frequency Stimulation at the Subiculum for Temporal Lobe Epilepsy Treatment in Rats. Neurobiol. Dis..

[38] de Oliveira J.C., de Castro Medeiros D., e Rezende G.H., Moraes M.F., Cota V.R. (2014). Temporally Unstructured Electrical Stimulation to the Amygdala Suppresses Behavioral Chronic Seizures of the Pilocarpine Animal Model. Epilepsy Behav..

[39] Mihály I., Orbán-Kis K., Gáll Z., Berki Á.J., Bod R.B., Szilágyi T. (2020). Amygdala Low-Frequency Stimulation Reduces Pathological Phase-Amplitude Coupling in the Pilocarpine Model of Epilepsy. Brain Sci..

[40] Yamamoto J., Ikeda A., Kinoshita M., Matsumoto R., Satow T., Takeshita K., Matsuhashi M., Mikuni N., Miyamoto S., Hashimoto N. (2006). Low-Frequency Electric Cortical Stimulation Decreases Interictal and Ictal Activity in Human Epilepsy. Seizure.

[41] Herrington T.M., Cheng J.J., Eskandar E.N. (2016). Mechanisms of Deep Brain Stimulation. J. Neurophysiol..

[42] Ashkan K., Rogers P., Bergman H., Ughratdar I. (2017). Insights into the Mechanisms of Deep Brain Stimulation. Nat. Rev. Neurol..

[43] Reddy D.S., Kuruba R. (2013). Experimental Models of Status Epilepticus and Neuronal Injury for Evaluation of Therapeutic Interventions. Int. J. Mol. Sci..

[44] Gimenes C., Moraes J., Battapady H., Tannus A., Federal U., Paulo D.S., Paulo S. (2019). The Neural Response to Deep Brain Stimulation of the Anterior Nucleus of the Thalamus: A MEMRI and c-Fos Study. Brain Res. Bull..

[45] Ghafouri S., Fathollahi Y., Semnanian S., Shojaei A., Asgari A., Amini A.E., Mirnajafi-Zadeh J. (2019). Deep Brain Stimulation Restores the Glutamatergic and GABAergic Synaptic Transmission and Plasticity to Normal Levels in Kindled Rats. PLoS ONE.

[46] Ladas T.P., Chiang C.C., Gonzalez-Reyes L.E., Nowak T., Durand D.M. (2015). Seizure Reduction through Interneuron-Mediated Entrainment Using Low Frequency Optical Stimulation. Exp. Neurol..

[47] Amorim B.O., Covolan L., Ferreira E., Brito J.G., Nunes D.P., de Morais D.G., Nobrega J.N., Rodrigues A.M., de Almeida A.C., Hamani C. (2015). Deep Brain Stimulation Induces Antiapoptotic and Anti-Inflammatory Effects in Epileptic Rats. J. Neuroinflamm..

[48] Miranda M.F., Hamani C., de Almeida A.C., Amorim B.O., Macedo C.E., Fernandes M.J., Nobrega J.N., Aarão M.C., Madureira A.P., Rodrigues A.M. (2014). Role of Adenosine in the Antiepileptic Effects of Deep Brain Stimulation. Front. Cell. Neurosci..

[49] Zhu G., Meng D., Chen Y., Du T., Liu Y., Liu D., Shi L., Jiang Y., Zhang X., Zhang J. (2018). Anterior Nucleus of Thalamus Stimulation Inhibited Abnormal Mossy Fiber Sprouting in Kainic Acid-Induced Epileptic Rats. Brain Res..

[50] Tang W., He X., Feng L., Liu D., Yang Z., Zhang J., Xiao B., Yang Z. (2020). The Role of Hippocampal Neurogenesis in ANT-DBS for LiCl-Pilocarpine-Induced Epileptic Rats. Stereotact. Funct. Neurosurg..

[51] Lüttjohann A., Fabene P.F., van Luijtelaar G. (2009). A Revised Racine’s Scale for PTZ-Induced Seizures in Rats. Physiol. Behav..

[52] Paxinos G., Watson C., Paxinos G., Watson C. (2007). The Rat Brain in Stereotaxic Coordinates.

[53] Khan A.A., Shekh-Ahmad T., Khalil A., Walker M.C., Ali A.B. (2018). Cannabidiol Exerts Antiepileptic Effects by Restoring Hippocampal Interneuron Functions in a Temporal Lobe Epilepsy Model. Br. J. Pharmacol..

[54] Kaminski R.M., Rogawski M.A., Klitgaard H. (2014). The Potential of Antiseizure Drugs and Agents that Act on Novel Molecular Targets as Antiepileptogenic Treatments. Neurotherapeutics.

[55] Fisher R.S., Velasco A.L. (2014). Electrical Brain Stimulation for Epilepsy. Nat. Rev. Neurol..

[56] Zhang S.H., Sun H.L., Fang Q., Zhong K., Wu D.C., Wang S., Chen Z. (2009). Low-Frequency Stimulation of the Hippocampal CA3 Subfield Is Anti-Epileptogenic and Anti-Ictogenic in Rat Amygdaloid Kindling Model of Epilepsy. Neurosci. Lett..

[57] André V., Marescaux C., Nehlig A., Fritschy J.M. (2001). Alterations of Hippocampal GABAergic System Contribute to Development of Spontaneous Recurrent Seizures in the Rat Lithium-Pilocarpine Model of Temporal Lobe Epilepsy. Hippocampus.

[58] Marx M., Haas C.A., Häussler U., Wierenga C.J. (2013). Differential Vulnerability of Interneurons in the Epileptic Hippocampus. Front. Cell. Neurosci..

[59] Kuruba R., Hattiangady B., Parihar V.K., Shuai B., Shetty A.K. (2011). Differential Susceptibility of Interneurons Expressing Neuropeptide Y or Parvalbumin in the Aged Hippocampus to Acute Seizure Activity. PLoS ONE.

[60] Long L., Xiao B., Feng L., Yi F., Li G., Li S., Mutasem M.A., Chen S., Bi F., Li Y. (2011). Selective Loss and Axonal Sprouting of GABAergic Interneurons in the Sclerotic Hippocampus Induced by LiClPilocarpine. Int. J. Neurosci..

[61] Wittner L., Erőss L., Szabó Z., Tóth S., Czirják S., Halász P., Freund T.F., Maglóczky Z.S. (2002). Synaptic Reorganization of Calbindin-Positive Neurons in the Human Hippocampal CA1 Region in Temporal Lobe Epilepsy. Neuroscience.

[62] Wittner L., Maglóczky Z. (2017). Synaptic Reorganization of the Perisomatic Inhibitory Network in Hippocampi of Temporal Lobe Epileptic Patients. BioMed Res. Int..

[63] Ellender T.J., Raimondo J.V., Irkle A., Lamsa K.P., Akerman C.J. (2014). Excitatory Effects of Parvalbumin-Expressing Interneurons Maintain Hippocampal Epileptiform Activity via Synchronous Afterdischarges. J. Neurosci..

[64] Wittner L., Erőss L., Czirják S., Halász P., Freund T.F., Maglóczky Z. (2005). Surviving CA1 Pyramidal Cells Receive Intact Perisomatic Inhibitory Input in the Human Epileptic Hippocampus. Brain.

[65] Fuentealba P., Begum R., Capogna M., Jinno S., Márton L.F., Csicsvari J., Thomson A., Somogyi P., Klausberger T. (2008). Ivy Cells: A Population of Nitric-Oxide-Producing, Slow-Spiking GABAergic Neurons and Their Involvement in Hippocampal Network Activity. Neuron.

[66] Capogna M. (2011). Neurogliaform Cells and Other Interneurons of Stratum Lacunosum-Moleculare Gate Entorhinal-Hippocampal Dialogue. J. Physiol..

[67] Beamer E., Otahal J., Sills G.J., Thippeswamy T. (2012). Nw-Propyl-l-Arginine (L-NPA) Reduces Status Epilepticus and Early Epileptogenic Events in a Mouse Model of Epilepsy: Behavioural, EEG and Immunohistochemical Analyses. Eur. J. Neurosci..

[68] González-Hernández T., García-Marín V., Pérez-Delgado M.M., González-González M.L., Rancel-Torres N., González-Feria L. (2000). Nitric Oxide Synthase Expression in the Cerebral Cortex of Patients with Epilepsy. Epilepsia.

[69] Sardo P., Ferraro G. (2007). Modulatory Effects of Nitric Oxide-Active Drugs on the Anticonvulsant Activity of Lamotrigine in an Experimental Model of Partial Complex Epilepsy in the Rat. BMC Neurosci..

[70] Carreira B.P., Santos D.F., Santos A.I., Carvalho C.M., Araujo I.M. (2015). Nitric Oxide Regulates Neurogenesis in the Hippocampus Following Seizures. Oxid. Med. Cell. Longev..

[71] Lumme A., Soinila S., Sadeniemi M., Halonen T., Vanhatalo S. (2000). Nitric Oxide Synthase Immunoreactivity in the Rat Hippocampus after Status Epilepticus Induced by Perforant Pathway Stimulation. Brain Res..

[72] Park C., Kang M., Kang K., Lee J., Kim J., Yoo J., Ahn H., Huh Y. (2001). Differential Changes in Neuropeptide Y and Nicotinamide Adenine Dinucleotide Phosphate-Diaphorase-Positive Neurons in Rat Hippocampus after Kainic Acid-Induced Seizure. Neurosci. Lett..

[73] Sloviter R.S., Zappone C.A., Harvey B.D., Bumanglag A.V., Bender R.A., Frotscher M. (2003). Dormant Basket Cell Hypothesis Revisited: Relative Vulnerabilities of Dentate Gyrus Mossy Cells and Inhibitory Interneurons after Hippocampal Status Epilepticus in the Rat. J. Comp. Neurol..

[74] Jinno S., Klausberger T., Marton L.F., Dalezios Y., Roberts J.D., Fuentealba P., Bushong E.A., Henze D., Buzsaki G., Somogyi P. (2007). Neuronal Diversity in GABAergic Long-Range Projections from the Hippocampus. J. Neurosci..

[75] Sun C., Mtchedlishvili Z., Bertram E.H., Erisir A., Kapur J. (2007). Selective Loss of Dentate Hilar Interneurons Contributes to Reduced Synaptic Inhibition of Granule Cells in an Electrical Stimulation-Based Animal Model of Temporal Lobe Epilepsy. J. Comp. Neurol..

[76] Arabadzisz D., Antal K., Parpan F., Emri Z., Fritschy J.M. (2005). Epileptogenesis and Chronic Seizures in a Mouse Model of Temporal Lobe Epilepsy Are Associated with Distinct EEG Patterns and Selective Neurochemical Alterations in the Contralateral Hippocampus. Exp. Neurol..

[77] Wang X., Song X., Wu L., Nadler J.V., Zhan R.Z. (2016). Persistent Hyperactivity of Hippocampal Dentate Interneurons after a Silent Period in the Rat Pilocarpine Model of Epilepsy. Front. Cell. Neurosci..

